# Severe asthma: a great clinical challenge

**DOI:** 10.1186/2045-7022-5-S2-P4

**Published:** 2015-03-23

**Authors:** Tanja Grzetic-Romcevic, Boris Devcic, Silvana Sonc

**Affiliations:** 1 Hospital Sezana, Sezana, Slovenia; 2 Hospital Sezana, Pulmonary Department, Sezana, Slovenia

## Background

Patients with severe asthma represent a significant unmet clinical need. At present, the underlying mechanisms of severe asthma have not been established yet but it is likely they reflect a heterogenous pattern rather than a single unifying process. The aim of this study is to assess the prevalence of severe asthma as well as the clinical and physiologic characteristics of patients and to establish a pattern occuring in comorbid diseases.

## Method

Between the January and December of 2013, a total of 324 consecutive asthmatic adult patients (67% females) attended to at an out - patients clinic (Sezana) were involved in the study. The mean age in these patients was 57.3 +- 2.4 years. The diagnosis of severe asthma was assessed according to the International ERS/ATS guidelines. The demographic criteria, body mass index BMI (kg/m2), skin prick tests (Allergopharma, Germany), smoking habits and comorbidities were analysed.

## Results

Severe asthma was present in 10% of patients. The mean age in patients was 67.3+- 1.7 years (59%females). Of them, 14% were active smokers and 24% were former smokers. Sixty- five percent patients had a positive family history of asthma. The average mean time of asthma duration was 14+- 3.2 years. The late onset of asthma (patients above 50 years of age) was observed in 60% of all cases. Atopy, which was estimated on the basis of at least one positive skin prick test, was observed in 66% of patients. In patients showing early onset, severe asthma involved significantly more allergen sensitivity (skin test positivity, 92% vs 26% p<0.01) than in patients with late- onset asthma. The average body mass index was 32.1+- 4.7 kg/m2. The obese patients were more frequently females (60% vs 40%). The comorbid diseases were present in 70% of patients. The most frequent were the comorbid conditions of the upper airways - nasal sinus diseases (54% of patients), mostly in females (44%). Next, there were aspirin sensitivity in females (10%) and OSA (obstructive sleep apnea) in males (10%). Both females and males suffered from GERD (gastroesophageal reflux disease) (15% males vs 10% females), and psychological dysfunction (82% females vs 61% males).

## Conclusion

The above results suggest than an adult asthmatic with severe asthma has a positive family history of asthma as well as a late onset of disease with less atopy by skin test and is also more obese (especially females). The prevalence of comorbidites is very high.

